# Microstream capnography during conscious sedation with midazolam for oral surgery: a randomised controlled trial

**DOI:** 10.1038/bdjopen.2017.19

**Published:** 2017-10-13

**Authors:** Paul Brady, Gabriella Iohom, Ken D O’Halloran, Christine McCreary, Michael Cronin

**Affiliations:** 1 Department of Dental Surgery, University College Cork, Cork, Ireland; 2 Department of Anaesthesia, University College Cork, Cork, Ireland; 3 Department of Physiology, University College Cork, Cork, Ireland; 4 Department of Statistics, University College Cork, Cork, Ireland

## Abstract

**Objectives/Aims:**

There has been no dentistry-specific published data supporting the use of monitoring with capnography for dental sedation. Our aim was to determine if adding capnography to standard monitoring during conscious sedation with midazolam would decrease the incidence of hypoxaemia.

**Materials and Methods:**

A randomised controlled trial was conducted in which all patients (ASA I and II) received standard monitoring and capnography, but were randomised to whether staff could view the capnography (intervention) or were blinded to it (control). The primary outcome was the incidence of hypoxaemia (SpO_2_⩽94%).

**Results:**

We enrolled 190 patients, mean age 31 years (range, 14–62 years). There were 93 patients in the capnography group and 97 in the control group. The mean cumulative dose of midazolam titrated was 6.94 mg (s.d., 2.31; range, 3–20 mg). Six (3%) patients, three in each group, required temporary supplemental oxygen. There was no statistically significant difference between the capnography and control groups for the incidence of hypoxaemia: 34.4 vs 39.2% (*P*=0.4962, OR=0.81, 95% CI: 0.45–1.47).

**Conclusions:**

We were unable to confirm an additive role for capnography to prevent hypoxaemia during conscious sedation with midazolam for patients not routinely administered supplemental oxygen.

## Introduction

For the routine provision of intravenous conscious (moderate) sedation, dentists typically use a monotherapy technique consisting of boluses of midazolam titrated to effect.^1^ Care should be taken to slowly titrate the drug to the desired end point, as there can be a wide variation in individual response. Most sedative and anaesthetic agents suppress respiratory drive; therefore, hypoxaemia remains the one factor that, if not carefully monitored and controlled, has the potential to cause serious complications. Current applicable guidelines recommend clinical and electromechanical monitoring including continuous pulse oximetry.^2^

In relation to capnography, Standards for Conscious Sedation in the Provision of Dental Care (2015) states the following:

*‘Despite the growing pressure for it to be used universally in the UK, until results of dentistry-specific research are available, its routine use for ASA grade 1 and 2 dental patients lacks high level scientific validation and cannot be recommended’*.^2^

Conversely, in North America and Australia, guidelines recommend capnography for moderate sedation.^3–5^ Microstream capnography, a relatively new technology for carbon dioxide (CO_2_) monitoring in exhaled air, is capable of reliably monitoring respiratory patterns in spontaneously breathing patients.^6^ A special nasal–oral cannula is used to continuously sample CO_2_ concentration in exhaled breath. From these measurements, the device plots an ongoing waveform and automatically calculates the maximum CO_2_ concentration in each breath, the end tidal carbon dioxide (EtCO_2_).

Previous research and two meta-analyses found that capnography reduced the incidence of hypoxaemia during procedural sedation.^7–11^ On the other hand, recent studies from gynaecological and endoscopy settings found capnography monitoring did not reduce the incidence of hypoxaemia.^12,13^ Therefore, the generalisability of published research on this issue is problematic. The lack of a uniform definition of hypoxaemia makes comparison across the studies difficult. This variability is further compounded by differences in patient populations, sedation settings, the use of supplemental oxygen and type of sedation agents and techniques. The aim of this study is to determine if the addition of microstream capnography to standard monitoring results in a decreased incidence of hypoxaemia during conscious sedation.

## Materials and methods

### Study design and setting

This was a prospective, parallel group, randomised controlled trial on patients receiving intravenous conscious sedation with midazolam for minor oral surgery procedures. The trial took place at Cork University Dental School and Hospital in Ireland between January 2014 and June 2015.

### Population

The study protocol was reviewed and approved by the Clinical Research Ethics Committee of the Cork teaching hospitals. Demographic details, including alcohol consumption and smoking, were recorded on the patient medical questionnaire. We excluded patients with ASA classes III–V, body mass index >35 kg/m^2^ and patients who refused or were unable to give informed consent. Written informed consent was obtained from 190 patients aged between 14 years and 62 years attending for minor oral surgery procedures ranging from single tooth removal to the surgical removal of impacted wisdom teeth. The study was conducted in accordance with the moral, ethical and scientific principles governing clinical research in the Declaration of Helsinki (2008) and Good Clinical Practice. The trial was registered at ClinicalTrials.gov under identifier NCT01949012.

### Study protocol

This study was specifically designed to evaluate the use of capnography monitoring for the most commonly used intravenous conscious sedation technique in primary dental care. The sedation team consisted of a dentist who administered the sedation (sedationist) and carried out the operative procedure (operator), and a dental nurse. This sedationist-operator model with a suitably trained dental nurse acting as the second appropriately trained person is common in dental sedation. The research team consisted of a research nurse and an independent researcher, both of whom had no direct involvement with the administration of sedation or the operative procedure. The researcher had received training on the interpretation of capnography and his role was to prompt the patient to take breaths in response to capnography readings only in the capnography group.

**Figure 1:**
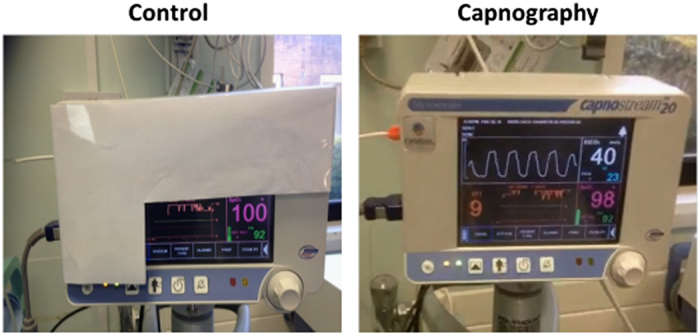
Vital signs monitor. Control (blinded capnography) and Capnography (intervention).

The randomisation choice was concealed from the teams until after enrolment. Randomisation was in blocks of varying length (two, four or six), generated using SAS version 9.3. Subjects were randomly assigned by the research nurse via sequentially numbered, sealed, opaque envelopes in a 1:1 ratio into one of two groups: the control group where the principal investigator and the treating team were blinded to the capnography screen, and the capnography (intervention) group where the capnography screen was viewable by all staff and the principal investigator (Figure 1). Participants and clinicians were aware of the treatment allocation by the time sedation was administered. In accordance with recognised guidelines, all patients were continuously monitored for heart rate, pulse oximetry, visual signs of breathing and periodic (at 10 min intervals) arterial blood pressure measurement.^1^ Midazolam was incrementally titrated at a rate of 1 mg/min to an end point determined by the dentist administering the sedation. Signs of adequate sedation included slurring of speech and slowed reaction to commands. All patients in the study had local anaesthetic (2% lignocaine with adrenaline) administered to provide analgesia for the procedure.

To enable sampling for microstream capnography, all the patients were fitted with a specialised nasal cannula oxygen device with a built-in sampling port (Smart Capnoline plus O_2_; Covidien Inc., Mansfield, MA) designed to capture inspired and expired carbon dioxide orally and nasally. The sampling port is specifically designed to work with standard oxygen delivery systems to enable the administration of supplemental oxygen when required. This sampling device was connected to the capnography monitoring device (Capnostream 20, Covidien, Mansfield, MA), which displayed real-time CO_2_ in waveform and numeric form, respiratory rates and pulse oximetry functions. Supplemental oxygen was not routinely administered during the sedations. In the capnography group, an independent research observer monitored the patients for respiratory activity via the capnography monitor. Any sign of apnoea, altered ventilation or oxygen desaturation to SpO_2_⩽94% prompted an intervention in a step-wise fashion that included verbal stimulation to take breaths and, if required, oxygen supplementation. The reversal of sedation with the benzodiazepine antagonist, flumazenil, was included in the protocol; however, sedation is not routinely reversed in conscious dental sedation. Indications for the administration of flumazenil include when safe saturation levels cannot be maintained and in a medical emergency.

Capnography waveforms on the capnography monitor enable visual assessment of ventilation. The capnographic criteria for respiratory depression or airway obstruction included a capnogram depicting alveolar hypoventilation, a flat waveform (apnoea) or a respiratory rate less than 10 breaths/min.

The capnostream 20 records data electronically every 2 s during the course of each sedation. Continuous data from the monitor was acquired on a laptop computer via a communications interface adapter employing specific oximetry software (Profox Associates inc. USA). This allowed for *post hoc* analysis of the monitoring data.

The majority of dental sedation is administered in primary care and the limitations of this environment must be appreciated.^14^ Therefore, early warning of desaturation is essential. The primary outcome variable was any occurrence of hypoxaemia defined as oxygen saturation (SpO_2_) ⩽94% of any duration. For the purposes of this study in a dental setting, safe oxygen saturation levels were defined as 95% and higher. When SpO_2_ drops to ⩽94%, it is a trigger for the sedation team to ask for breaths. The oxyhaemoglobin desaturation curve is sigmoidal, and desaturation occurs very rapidly in the apnoeic or airway-obstructed patient once levels of 92% and lower are reached. The secondary outcome was the degree of hypoxaemia.

### Statistical analysis

The sample size calculation was based on (1) an observation study investigating capnometry that was performed in a sample of 33 adults in the same setting at this institution and (2) studies from other settings that demonstrated levels of hypoxaemia between 17 and 30%.^15–17^ A sample size of 95 per group had 80% power at the 5% level of significance to demonstrate a reduction in proportions of patients experiencing any hypoxaemia (SpO_2_⩽94%) from 30% in the control group to 15% in the capnography group. The primary outcome measure of any hypoxaemia (SpO_2_⩽94%) was compared between the capnography and control groups using logistic regression comparisons and *x*^*2*^ test of proportions in SAS (Version 9.4). Analyses were performed on the following secondary outcome measures: the proportion of patients experiencing moderate hypoxaemia (defined as 90%⩽SpO_2_⩽92%) and the proportion of patients experiencing severe hypoxaemia (defined as SpO_2_<90%). Interventions to correct hypoxaemia in response to desaturation were quantified as the number of patients receiving verbal prompts to take breaths and the number of patients receiving supplemental oxygen.

## Results

**Figure 2:**
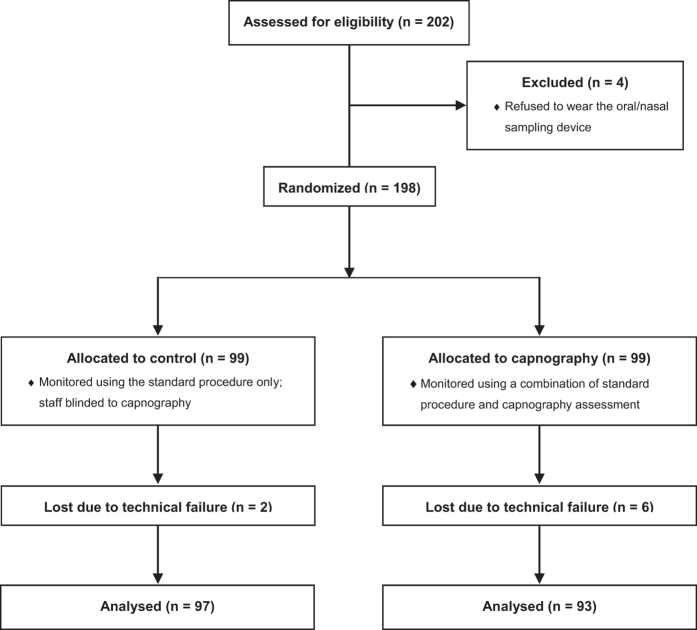
CONSORT recrutment diagram.

We screened 202 patients for possible study participation. Four refused to wear the oral/nasal cannula prior to the randomisation envelope being opened; the remaining 198 patients were randomised to the control or the capnography (intervention) group. Eight patients were removed during data processing as more than 20% of their pulse oximetry or capnography data was missing or incomplete at *post hoc* analysis. Technical difficulties, such as recording errors and patient movement, were the most common reasons for the loss of data. There were 190 patients in the analysis: 97 in the control group and 93 in the capnography group (Figure 2).

### Outcome measures

For the primary outcome measure, we found no difference in the proportion of patients who experienced hypoxaemia (SpO_2_⩽94%) between the capnography and the control group 34.4 vs. 39.2% (*P*=0.4962, OR=0.81, 95% CI: 0.45–1.47). The number of patients receiving verbal stimulation to take breaths was higher in the capnography group than in the control group: 54 vs 38 (Table 1). The mean cumulative dose of midazolam per patient at end point was 6.94 mg (s.d., 2.31) and the range of dosage 3–20 mg.

Six (3%) patients, three in each group, required supplemental oxygen. The mean age of patients requiring supplemental oxygen was 48.83 years (s.d., 13.89). Their mean cumulative dose of midazolam at end point was 5.6 mg (s.d., 1.3) and the dosage range was 4–8 mg.

## Discussion

We were unable to demonstrate that the addition of capnography to standard clinical and electromechanical monitoring for moderately sedated (conscious) patients on room air results in a decreased incidence of desaturation in an oral surgery setting.

Conversely, a meta-analysis revealed that respiratory depression was 17.6 times more likely to be detected during procedural sedation in patients who were monitored with capnography than with standard monitoring alone.^10^ Many of the studies in this meta-analysis were from settings where deep sedation was the target: supplemental oxygen was routinely administered and combinations of drugs were used. In the United Kingdom and Ireland, sedation in the deep end of the sedation continuum is regarded as general anaesthesia and falls outside the definition of moderate (conscious) sedation.^1^ In the present study, we utilised conscious sedation and patients were not pre-oxygenated with supplemental oxygen. Our results are in agreement with recently published data from two clinical trials in endoscopy and gynaecology settings, which were unable to confirm an additive role for capnography in preventing hypoxaemia for patients not receiving routine supplemental oxygen.^12,13^ Our findings are also in agreement with a study from an emergency room setting evaluating whether capnography or pulse oximetry first detected respiratory events in sedated adults breathing room air.^18^ They found pulse oximetry detected hypoxaemia well before capnographic changes were observed. The term procedural sedation includes moderate and deep sedation on the sedation continuum. Perhaps, when considering the evidence for capnography monitoring, the term procedural sedation is too broad. Certainly, for deep sedations where supplemental oxygen is required, the evidence supports capnography monitoring to reduce the incidence of hypoxaemia.^7–11^ However, for conscious sedation where patients breathe room air, it would appear from our findings and also from other studies that the addition of capnography does not significantly reduce the incidence of hypoxaemia.^12,13^

Our dosage illustrates the wide range when titrating midazolam to effect in this setting. We chose an oxygen saturation of ⩽94% as the primary outcome measure, in part because it represents a consensus threshold point in dental sedation units at which staff acts to stimulate a sedated patient out of concern that the patient is not adequately ventilating. In the present study, the overall level of hypoxaemia (SpO_2_⩽94%) was 37%, which is consistent with the reported incidences in reasonably comparable studies that demonstrated levels of hypoxaemia between 17 and 47% with midazolam.^17,18^ Just 10% of our patients had an SpO_2_ event ⩽90%. An SpO_2_ of 90% is the recommended setting for alarm activation on pulse oximeters for dental sedation.

Females are known to be more dentally anxious than males.^19^ In the present study, as shown in Table 2, there were over twice as many females as males enrolled (131 females and 59 males).

In accordance with our institutional policy, patients did not routinely receive supplemental oxygen unless indicated due to hypoxaemia. The percentage of patients requiring supplemental O_2_ is comparable with a similar previous study in an emergency room setting.^20^ Routine supplemental O_2_ administration is considered to diminish the value of pulse oximetry as a respiratory monitor of early respiratory compromise, since hypoxaemia becomes a late sign of respiratory depression.^21^ Deitch *et al*.^22^ found that supplemental O_2_ was related to a higher incidence of respiratory depression. More patients were stimulated to take breaths in our capnography group than in the control group. It is likely that many of the capnography changes seen in the present study were subclinical. Episodes of respiratory depression occurring during sedation can be transient and not all such occurrences result in patient harm.^9^ In this context, it is important to note that calling for breaths in response to transient respiratory depression that would have resolved spontaneously without harming the patient may be counterproductive because it could result in inadequate sedation.^23^ Interestingly, Barnett *et al*.^24^ found that patients in their capnography group experienced significantly more discomfort. They hypothesised that this may relate to interventions (e.g., airway repositioning) in response to clinically insignificant EtCO_2_ waveform changes that result in patients being more frequently stimulated. None of our patients required reversal of the sedation with the benzodiazepine antagonist flumazenil and all patients had their treatment successfully completed.

The results of this study can only be applied to situations where midazolam is administered as a single drug for conscious sedation in healthy adults. The importance of patient selection and pre-sedation assessment cannot be overemphasised to ensure a healthy cohort of patents for sedation in a dental setting who are unlikely to require supplemental oxygen. The key principle is that when supplemental oxygen is provided for any reason, the pulse oximeter must never be misconstrued as monitoring ventilation. For patients requiring supplemental oxygen, monitoring with capnography will provide the clinician with useful additional information regarding respiratory function. Capnography should be taught on dental sedation courses and we recommend a move towards having capnography at hand for situations when supplemental oxygen is administered. Vital signs monitors with capnography functionality are becoming more affordable and clinicians purchasing new monitors should look for this function to be included. Benzodiazepines may be up to eight times more potent after prior administration of an opioid and respiratory depression is more likely to occur when more than one sedative agent is administered.^25,26^ With the growing usage of drugs in combination to deliver dental sedation, there is a need for future research as respiratory depression is more likely with drugs in combination than for the monotherapy technique in the present study.

### Limitations

The principal strength of this study is that it was conducted in a real-world setting albeit that an independent researcher was used for the capnography interpretation. There are several potential limitations to our study. First, it is possible that our continuous electronic data collection influenced the rate of hypoxaemia because it would detect episodes that might otherwise be missed through human inattention or error. Second, while obvious artifacts were eliminated during analysis of the recorded data, the possibility of residual miscalculation of hypoxaemia due to artifacts remains. Third, we did not record the depth of sedation by using a scale, such as the Ramsey scale, or by monitoring with bispectral index or entropy. We believe that the randomisation process would have led to equitable distributions of depth of sedation between the two groups. Thus, recording the depth of sedation would not have had any significant impact on the overall study results. Future research should evaluate the impact of capnography on patient satisfaction during routine oral surgery.

## Conclusion

This study set out to determine if adding capnography to standard monitoring during conscious sedation would decrease the frequency of oxygen desaturation in an oral surgery setting. We were unable to confirm an additive role for capnography to prevent hypoxaemia during conscious sedation with midazolam (monotherapy) in ASA I and II patients not routinely administered supplemental oxygen. The results of this study provide dentistry-specific evidence to support current guidelines with regard to monitoring with capnography.

## Figures and Tables

**Table 1:** Primary and secondary outcome measures: capnography versus control groups

	*Capnography (*n*=93)*	*Control (*n*=97)*	P-*value*	*Odds ratio*	*95% CI*	
*Primary outcome*						
Patients with any hypoxaemia	34.4%	39.2%	0.4962	0.814	0.451	1.471
						
*Secondary outcomes*						
Patients with:						
Moderate hypoxaemia	24.7%	23.7%	0.8697	1.057	0.544	2.053
Severe hypoxaemia	11.8%	8.3%	0.4132	1.492	0.572	3.893
Patients receiving:						
Verbal stimulation	54	38	0.0139			
In response to SpO_2_	*17*	*38*				
In response to capnography	*37*	*—*				
Supplemental O_2_	3	3	0.8236			

Logistic regression comparisons and χ^2^-test of proportions. Any (SpO_2_≤94%), moderate (90%≤SpO_2_≤92%) and severe (SpO_2_<90%) hypoxaemia.

**Table 2:** Characteristics of capnography and control arms for enrolled patients

*Characteristic*	*Capnography (*n*=93)*	*Control (*n*=97)*	P *value*
	*n (%)*		
*Gender*			
Male	24 (26%)	35 (36%)	0.13
Female	69 (74%)	62 (64%)	
			
Alcohol drinker	59 (63%)	52 (54%)	0.23
Smoker	24 (26%)	26 (27%)	0.88
			
*ASA classification*			
I	65 (70%)	61 (63%)	0.31
II	28 (30%)	36 (37%)	
			
	*Mean (s.d.)*		
Age (years)	31.2 (12.7)	31.5 (12.4)	0.86
BMI (kg/m^2^)	24.5 (3.7)	24.4 (4.2)	0.99
			
*Baseline parameters*			
EtCO_2_ (mm Hg)	37.6 (3.8)	36.4 (4.1)	
Respiratory rate (per min)	17.9 (4.6)	17.3 (4.5)	
SpO_2_ (%)	99.1 (1.2)	99.2 (1.2)	

Abbreviations: ASA, American Society of Anesthesiologists; BMI, body mass index; EtCO_2_, end-tidal carbon dioxide; SpO_2_, haemoglobin oxygen saturation.

Data are presented as mean (s.d.) or *n* (%) as appropriate.
